# Childhood Influenza Vaccination and Its Determinants during 2020–2021 Flu Seasons in China: A Cross-Sectional Survey

**DOI:** 10.3390/vaccines10121994

**Published:** 2022-11-23

**Authors:** Kaiyi Han, Zhiyuan Hou, Shiyi Tu, Qian Wang, Simeng Hu, Yuting Xing, Jing Du, Shujie Zang, Tracey Chantler, Heidi Larson

**Affiliations:** 1 School of Public and Health, Fudan University, Shanghai 200032, China; 2 Department of Infectious Disease Epidemiology, London School of Hygiene & Tropical Medicine, London WC1E 7HT, UK; 3 NHC Key Laboratory of Health Technology Assessment, Fudan University, Shanghai 200032, China; 4 Department of Global Health and Development, London School of Hygiene & Tropical Medicine, London WC1E 7HT, UK; 5 Department of Health Metrics Sciences, University of Washington, Seattle, WA 98195, USA

**Keywords:** influenza, vaccine, child, confidence, China

## Abstract

Young children aged 6–59 months are recommended as one of the priority groups for seasonal influenza vaccination in China. This study assessed influenza vaccination coverage and the factors associated with vaccination uptake among children in three Chinese provinces. In September 2021, 2081 caregivers with children <5 years completed self-administered questionnaires as part of a cross-sectional survey. Logistic regression was used to assess determinants of childhood influenza vaccination. A total of 43.63% of respondents reported vaccinating their children against influenza during the 2020–2021 flu season. Caregivers who lived in Anhui province, had a bachelor degree or above, and an annual household income <20,000 RMB were more likely to vaccinate their children against influenza. Confidence in the importance (OR: 2.50; 95%CI: 1.77–3.54), safety (OR: 1.60; 95%CI: 1.29–1.99), and effectiveness (OR: 1.54; 95%CI: 1.23–1.93) of influenza vaccine was significantly associated with childhood vaccine acceptance. Respondents who saw that other caregivers were vaccinating their children had significantly higher odds of vaccinating their own children. Caregivers’ receiving positive influence from healthcare workers (OR: 1.33; 95%CI: 1.00–1.77), family members, or friends (OR: 1.30; 95%CI: 1.14–1.49) were also significantly associated with childhood influenza vaccination. Poor access, including conflicts between caregivers’ availability and vaccination service schedules and inconvenient transportation to the vaccination site were negatively associated with childhood flu vaccination. To promote childhood influenza vaccination, public health information campaigns need to target wealthier and less educated caregivers to enhance caregivers’ confidence in influenza vaccination. Targeted interventions are also needed to optimize access to vaccination services, including extending vaccination service hours and increasing the number of vaccination sites close to residential areas. Interventions are also needed to encourage primary care providers to play a greater role in promoting vaccination. Finally, the dissemination of related information and the public response need to be monitored for the timely understanding of public perceptions.

## 1. Introduction

Seasonal influenza is an infection of the airways caused by influenza viral strains that undergo annual antigenic variation. Annually, influenza can affect 5% to 15% of the world’s population, with an estimated global burden of 3 to 5 million cases of severe disease and a death toll ranging from 290,000 and 650,000 [1]. Influenza viruses can cause disease in all age groups, but children have the highest rates of infection [2]. Limited studies suggest that the incidence of seasonal influenza in children is around 30% in China [3,4], causing a large economic burden [5].

The World Health Organization (WHO) recommends that children be a priority group for vaccination [6]. Over 40% of countries offer free seasonal influenza vaccination in their National Immunization Schedules, including most countries in North and South America, Europe, and some countries in Africa, South-East Asia, and the West Pacific Region [7,8,9,10,11]. In China, the national influenza vaccination coverage for all ages is only 2.2%, recorded in 2014 [12], and data collected between 2009 and 2012 indicate that influenza vaccination uptake among children <5 years living in five provinces in mainland China was about 26.4% [13]. Several factors contribute to this low coverage. Firstly, the seasonal influenza vaccine is not included in the national expanded program on immunization (EPI) and needs to be paid for out of pocket, and secondly, previous studies have focused on the influence of individual-level knowledge, attitudes, and beliefs on vaccination behavior. Results showed that caregivers’ (i.e., parents and guardians) have a poor awareness of risk, fear of adverse reactions after vaccination, and poor knowledge of influenza, which are major obstacles to increase the childhood influenza vaccination coverage [14,15,16].

However, there are limitations to existing studies. Firstly, existing studies that adapted the health belief model (HBM) largely focused on individual perceptions and did not investigate external factors. Access to vaccination services and broader areas of fit between the patient and the healthcare system, which includes availability, accessibility, accommodation, affordability, and acceptability, were not considered [17]. Secondly, the HBM was developed based on the assumption that humans are rational decision-makers [13]. However, even when individuals have comprehensive information and make choices with the intention of maximizing utility, they do not exclusively act rationally [18]. Emotions aroused by getting or not getting vaccinated can influence vaccination decision-making; however, most existing HBM studies do not account for these factors. Hence, we argue that these factors need to be included to improve the predictive power of the model. Thirdly, multiple information sources play an increasingly important role in decision-making, and access to appropriate information is essential to guide vaccination decisions [19]. Different information sources vary in their reliability and impact on the individual. For example, health professionals are regarded as the most reliable information sources [20], while information from media and interpersonal sources may be ambiguous [21,22].

More importantly, the COVID-19 pandemic, which had an unrivalled impact on global healthcare and social systems, may also affect public perception and attitudes toward influenza vaccines. Though the COVID-19 virus and influenza are vastly different pathogens, there are important areas of overlap [23]. For example, the majority of COVID-19 patients present with influenza-like illness [24]. Meanwhile, the adoption of nonpharmacologic interventions (NPIs), such as mandated face coverings in public, have influenced the incidence of influenza. Study showed decreased influenza incidence in 2020 (January through May) after adoption of NPIs as compared with prior seasons [25]. Thus, it is necessary to understand how public perceptions of influenza changed during COVID-19.

Our study aimed to provide updated estimates of the uptake level of influenza vaccination among children during COVID-19, and to assess the association not only between individual perceptions but also access to influenza vaccination, emotion of caregivers (i.e., parents, and guardians), and various information sources and childhood influenza vaccination.

## 2. Materials and Methods

### 2.1. Study Design

From September to November 2021, we conducted a cross-sectional survey in Shenzhen megacity in Guangdong province, Anhui province, and Shaanxi province, located in eastern, central, and western China, respectively. A two-stage cluster-sampling process was used to enroll eligible participants self-identified as parents or guardians of children <6 years old: (1) we selected three urban districts and two rural counties in the three provinces; (2) three or four communities were selected according to their socioeconomic status in each district/county. In each sampled community, caregivers were recruited from one vaccination clinic and one kindergarten, respectively. Caregivers of all children visiting the vaccination clinics on a given day during the survey period and from a class in the sampled kindergartens were invited to participate in the survey. Caregivers were invited to complete questionnaires by themselves after signing informed consent.

The Fudan University School of Public Health and the London School of Hygiene & Tropical Medicine Ethics committees approved the study protocol (FDU IRB#2018-10-0703, LSHTM Ethics Ref 160160).

### 2.2. Data Collection

A web-based questionnaire was developed using Questionnaire Star [26], a website that helps generate, distribute, and retrieve electronic questionnaires on a mobile platform. Respondents could access the questionnaire through WeChat, a Chinese social media platform with 1.1 billion active users. Each WeChat account was allowed to fill in the questionnaire once to avoid data duplication. The questionnaire was pilot tested in June 2021 among 10 respondents in a non-study community in Shanghai. It took approximately 3 min to complete the self-administered questionnaire; therefore, we used 3 min as a cutoff point for valid questionnaires. Respondents received electronic currency worth CNY 5 (US 0.7) as a gift after they completed the questionnaire. In total, 2877 caregivers accepted our invitation to participate in the survey. After excluding those completed in less than three minutes (256) and with children older than five years old (540). A total of 2081 respondents with valid data were included for analysis.

### 2.3. Instruments

The questionnaire included questions about demographic and socio-economic characteristics; perception of influenza vaccine and disease; cues to action, emotions, influence of various information sources; and acceptance of childhood influenza vaccination. Childhood influenza vaccination behavior was measured with the question, “Did your child receive influenza vaccine during the last flu season (October 2020 March 2021)?”. The questions used to measure the perception of influenza vaccine and disease and cue to action of caregivers and the corresponding scale are presented in Table A1 in Appendix A.

For perceived barriers, we investigated not only caregivers’ confidence in the importance and safety of influenza vaccines, but also the reasons for the lack of confidence. Regarding lack of confidence in vaccine importance, specific reasons include “It’s better to have natural immunity against influenza”, “Vaccines do not work (children still catch a cold after being vaccinated)”, “Flu is self-limiting for most people”, and “There is another useful treatment if my child gets flu”. Specific reasons for lack of confidence in vaccine safety include “The vaccine may give them flu”, “There will be side effects of the influenza vaccine”, “Child has allergy to chicken products” and “This flu vaccine would have a negative effect in interaction with other vaccines to be taken up by the child”. We also investigated caregivers’ vaccine confidence in general (effectiveness, safety and importance).

In addition, the emotions of caregivers were investigated by asking respondents if they ever felt worry, anxiety, or fear because they think that (1) not vaccinating with influenza vaccine will mean they get infected and (2) influenza vaccination will cause adverse reactions. Caregivers were also asked to comment on the role of social connections and institutions in their decision-making process: (1) health professionals; (2) friends or family members; (3) CDC or government department; and (4) internet or social media. Impacts of these different influencers include “recommended influenza vaccine” and “not recommended influenza vaccine”.

### 2.4. Statistical Analysis

Data from the online questionnaires were automatically uploaded to the Wenjuanxing online platform in real time. Descriptive analysis was used to describe the demographic and socio-economic characteristics, and caregivers’ perceptions on influenza and vaccines. The chi-square test was performed to compare the levels of influenza vaccine uptake with caregivers’ demographic and socio-economic characteristics and perception on influenza and vaccine and childhood influenza vaccination behavior. We used logistic regression analysis to identify the factors significantly associated with childhood influenza vaccine uptake. The associations are reported as odds ratios (ORs) with 95% confidence intervals (CIs). All tests were two-tailed, and *p*-values less than 0.05 were considered statistically significant. All statistical analyses were performed using Stata, version 14.0 (StataCorp LP, College Station, TX, USA).

## 3. Results

### 3.1. Sample Characteristics

Caregivers’ demographic and socio-economic characteristics are presented in Table A2 in Appendix B. A total of 2877 questionnaires were collected from caregivers. After excluding those completed in less than three minutes and completed by caregivers with children >6 years old, questionnaires from 2081 caregivers (72.33%) were included in the analyses. Of the 2081 respondents with valid data, 81.45% of caregivers’ residence is registered locally, and 62.37% lived in urban areas. Around 70% of caregivers were less than 35 years old, and 77.9% were mothers. Over half (54.45%) of caregivers had obtained junior college or bachelor level education or above, and 39.64% had an annual household income of less than CNY 50,000. Around 52% of caregivers’ children were male, and the majority (79.53%) were ≥24 months old.

### 3.2. Childhood Influenza Vaccination Behavior in 2020–2021 Influenza Season

**Figure 1:**
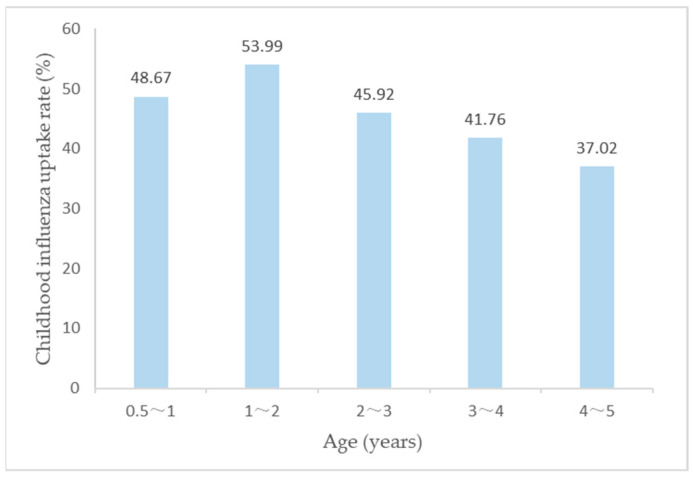
The Influenza vaccination uptake rates among children of each age group in three provinces.

Nearly half (43.63%) of the respondents reported that they vaccinated their children against influenza in the 2020–2021 flu season. Among children of different age groups, children aged 1–2 years had the highest uptake rate of 53.99%, while children aged 4–5 years had the lowest uptake rate of 37.02% (Figure 1).

### 3.3. Caregivers’ Perception on Influenza and Vaccine in 2020–2021 Influenza Season

**Figure 2:**
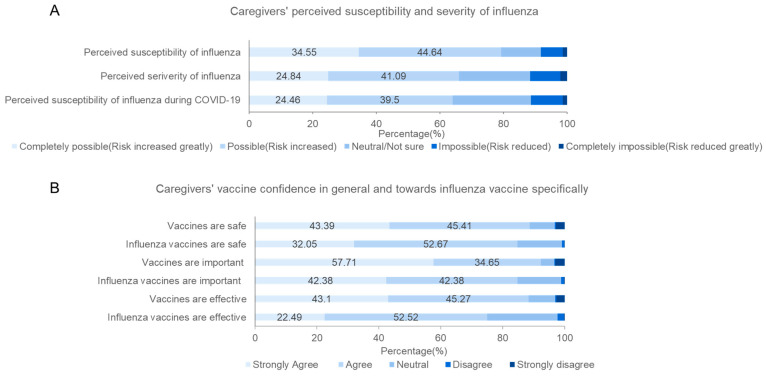
Caregivers’ perception on influenza and influenza vaccine: (**A**) Caregivers’ perceived susceptibility and severity of influenza; (**B**) Caregivers’ vaccine confidence in general and towards influenza vaccine specifically.

**Figure 3:**
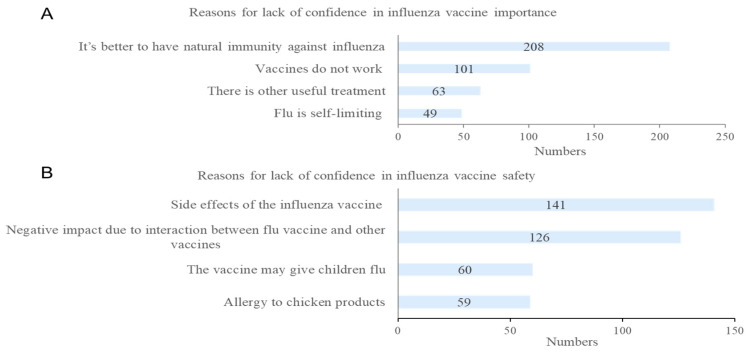
Caregivers’ reasons for lacking vaccine confidence: (**A**) Reasons for lack of confidence in influenza vaccine importance; (**B**) Reasons for lack of confidence in influenza vaccine safety.

The majority (79.19%) of caregivers agreed that their children are highly susceptible to influenza and 63.96% believed that the susceptibility increased during the COVID-19 pandemic (Figure 2). In addition, 65.93% agreed that the likelihood of serious health consequences after getting infected with influenza is high. Three-quarters (75.01%) of caregivers agreed that flu vaccines are effective with 84.77% and 84.72% agreeing that flu vaccines are important for children and safe, respectively, while 88.8%, 92.36%, and 88.37% agreed that vaccines are safe, important, and effective, respectively. The main reason for lack of confidence in the importance of influenza vaccine reported by 10.0% caregivers (*n* = 208) was believing that it is better to treat diseases through the child’s own immunity (Figure 3). Meanwhile, the top reasons cited by the respondents who were unsure about influenza vaccine safety were concerns about potential side effects in 6.78% (*n* = 141).

**Figure 4:**
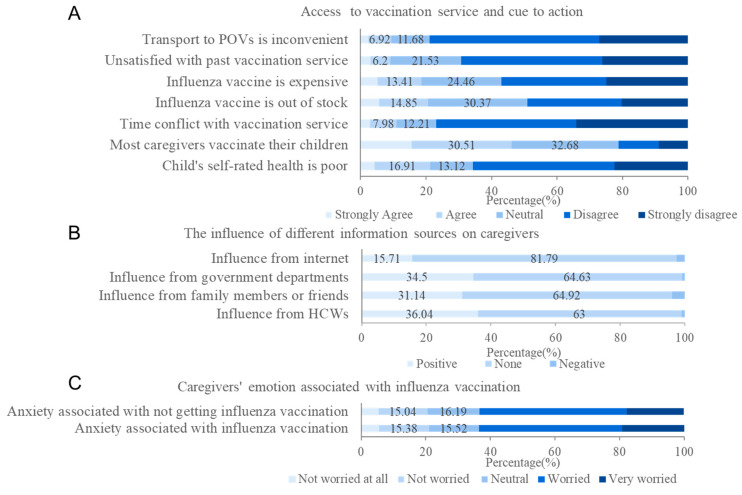
Access to vaccination service, influence of information sources, and caregivers’ emotion associated with influenza vaccination: (**A**) Access to vaccination service and cue to action; (**B**) The influence of different information sources on caregivers; (**C**) Caregivers’ emotion associated with influenza vaccination.

In terms of access to vaccination services, 10.91% agreed that the there was a conflict between their work and vaccination service schedules, with 20.57% and 18.6% agreeing that there were flu vaccine shortages and flu vaccines were expensive, respectively. Only 9.18% caregivers stated that they were unsatisfied with past vaccination service and 9.37% stated that transport to points of vaccination (POVs) was inconvenient (Figure 4).

The proportion of caregivers who reported that those who had a positive influence on their vaccine decisions included healthcare workers, family members or friends, government departments and the internet was 36.04%, 31.14%, 34.5%, and 15.71%, respectively. A total of 46.18% of caregivers stated that most of the parents they knew had their children vaccinated against the flu, and 21.24% think their children are in poor health. Moreover, 79% of caregivers had experienced anxiety about the safety of influenza vaccines, while 79.48% had anxiety because their children had not been vaccinated (Figure 4).

### 3.4. The Determinants of Childhood Influenza Vaccination

Table 1 show the univariate associations between childhood influenza vaccination and caregivers’ demographic and socio-economic characteristics and their perception on influenza and vaccines. The prevalence of childhood influenza vaccination varied significantly by demographic characteristics such as province, rural or urban area, children’s age, and socio-economic characteristics, such as caregiver education.

Childhood influenza vaccination was positively associated with a high level of caregivers’ perceived susceptibility and severity of flu. It was also significantly higher among those who have confidence in the importance, safety, and effectiveness of the flu vaccine; saw other caregivers vaccinating their children; received a positive influence from HCWs, family members or friends, government departments, and internet; and less anxiety associated with flu vaccination. Childhood influenza vaccination was negatively associated with caregivers’ poor access to the vaccination services including conflicts between work schedules and time of the vaccination service and inconvenient transport to the point of vaccination (POV).

After adjustment for covariates by multivariate logistic regression (Table 1). This analysis showed no relationship between childhood flu vaccination and other caregivers’ socio-economic characteristics, except for province (Shaanxi province: OR: 0.66; 95%CI: 0.48–0.92), education level (Bachelor degree or above: OR: 1.41; 95%CI: 1.13–1.76), and annual household income (100,000 to 200,000 RMB: OR: 0.81; 95%CI: 0.68–0.95; >200,000 RMB: OR: 0.73; 95%CI: 0.65–0.82). Confidence in the effectiveness (OR:1.54; 95%CI: 1.23–1.93), importance (OR: 2.50; 95%CI: 1.77–3.54), and safety (OR: 1.60; 95%CI: 1.29–1.99) of vaccines was positively associated with childhood flu vaccination. Poor access, including conflicts between caregivers’ schedules (OR: 0.61; 95%CI: 0.40–0.92) and vaccination service and inconvenient transportation to POV (OR: 0.66; 95%CI: 0.51–0.85) was negatively associated with childhood flu vaccination. In addition, caregivers who saw that other caregivers were vaccinating their children (OR: 2.16; 95%CI: 1.94–2.40) and were positively influenced by HCWs (OR: 1.33; 95%CI: 1.00–1.77), family members, or friends (OR: 1.30; 95%CI: 1.14–1.49) were more likely to vaccinate their children.

## 4. Discussion

Our study examined the factors associated with the childhood influenza vaccination uptake rate during the 2020–2021 flu season by drawing on cross-sectional survey data collected in three Chinese provinces. Our analyses found that the influenza vaccination uptake rate was 43.63% across all three provinces. Caregivers who lived in Anhui province, had a bachelor degree or above, and an annual household income <20,000 RMB were more likely to vaccinate their children against influenza compared to their counterparts. Having confidence in the importance of influenza vaccination and confidence in the vaccine effectiveness and safety were positively associated with childhood influenza vaccination. Convenient time of vaccination services, convenient transport to POVs, seeing that most parents around have had their children vaccinated against the flu, and getting a positive recommendation from HCWs and family members or friends were the key factors associated with caregivers being more likely to vaccinate their children in the 2020–2021 flu season.

Our analyses were similar to estimated childhood influenza vaccination uptake collected in Guangzhou in 2013 (47%) [16] but higher than the data collected in Qinghai Province (the 2014–2015 flu season: 11.4%; the 2015–2016 flu season: 11.9%) [15]. These findings show higher rates than the childhood influenza vaccination rate in Singapore (the 2015–2016 flu season: 32%) [27] and Thailand (the 2018–2019 flu season: 9%) [28]. It is also lower than that in other countries or regions, such as Hong Kong (the 2011–2012 flu season: 63.2%) [29,30], England (the 2015–2016 flu season: 52.8%) [31], and South Korea (the 2013–2014 flu season: 96.4%) [32]. Notably, our study showed a high vaccination uptake rate, even though influenza vaccines were not included in the national immunization program (NIP) in China, possibly due to the COVID-19 pandemic. The majority of COVID-19 patients in China presented with influenza-like illness (ILI), which increased the public awareness of respiratory pathogens [33]. Searches for the influenza vaccine during the 2020–2021 flu season were more frequent than previous ones [34], indicating increased public awareness about influenza vaccination.

Respondents’ confidence in the importance, effectiveness, and safety of influenza vaccines were independent predictors of childhood influenza vaccination in our analyses. The association between caregivers having a high level of vaccine confidence and their decision on vaccination is consistent with previous research, highlighting the importance of maintaining vaccine confidence [15,16]. As for caregivers’ confidence in all dimensions of the influenza vaccine, compared with the global average for vaccines confidence in general, our study showed a positive view on influenza vaccines’ safety (84.72% vs. 79% global), but more negative views on influenza vaccine effectiveness (75.01% vs. 84% global) [35]. Compared with vaccine confidence in general among caregivers, the proportions of respondents who had confidence in the safety of influenza vaccines and caregivers who agreed that vaccines in general were safe were similar, with them less likely to consider influenza vaccines as being effective and important. The gap in caregivers’ confidence in the importance and effectiveness of influenza vaccines versus vaccines in general is consistent with previous studies [36]. Our study also showed that the main reasons for lack of confidence in the importance and safety of vaccines reported by caregivers was believing that it is better to treat diseases through the child’s own immunity and having concerns about side effects, respectively. Therefore, targeted efforts should be made to address caregivers’ lack of confidence on vaccine importance and safety.

Delivering accurate and timely information about vaccines is a good way to increase caregivers’ confidence and promote vaccine acceptance [19]. The influence of different information sources, including health professionals, media, and interpersonal sources on the public’s attitude and decision on vaccines was inconsistent across countries [37]. To investigate the impact of various information sources on caregivers’ decisions about vaccines, we asked caregivers about the positive or negative impact of those sources on their decisions regarding childhood influenza vaccination. The percentage of caregivers who reported being positively influenced by healthcare workers was the highest because health professionals are regarded as the most reliable information sources [38]. We also found that caregivers who reported being positively influenced by healthcare workers were more likely to vaccinate their children, which is consistent with previous studies.

Evidence-based information from healthcare professionals could improve caregivers’ vaccination knowledge and positively influence awareness of the need for vaccination [39]. However, only 36.04% of caregivers reported positive influence from healthcare providers. The reason may lie in the low percentage (69%) of the public who refer to doctors as their primary source of information [37], which was 15–20% lower than estimates from developed countries [40,41]. In addition, caregivers commonly use the internet to search for information and guide their health-related decisions [42]. Although the internet makes it possible to overcome the spatial and temporal barriers to obtaining information 24/7, the quality of the information available online is questionable, with misinformation sometimes spreading more widely than positive and accurate information [37]. This could explain why only 15.71% of caregivers were positively affected by the internet. Barriers exist to increase Chinese caregivers’ access to vaccine information from professional sources. Firstly, there is a segmentation between clinical and preventive care in China. Vaccination services and related health education or consultations are provided by few dedicated vaccinators at POVs, while other primary care providers do not play an active role in it [43]. Hence, further research is needed to investigate the communication mode between HCWs and caregivers. Tailored interventions are needed to support and encourage primary care providers to play a greater role in promoting vaccination. Secondly, as exposure to misinformation may influence public perceptions of risk, and these risk perceptions are likely to be amplified through viral dissemination on the internet, the dissemination of related accurate information and the public response need to be monitored for timely understanding of public attitudes and perceptions [44].

When humans face risks, emotional responses, including feeling worry/anxiety might influence individuals’ decision-making process [45,46]. However, we didn’t find any significant associations between emotional responses to seasonal influenza and behavioral responses, which is not consistent with previous studies [16,47,48]. Further qualitative research is needed to explore the emotional responses experienced by caregivers during flu season and its impact on vaccination decision making. Previous studies in China have not focused on the access of vaccination services. Our study reported positive views on it. Only 10% of the respondents felt that they had a time conflict with the hours of vaccination service, were not satisfied with the vaccination service in the past, and felt the transportation to the POVs was inconvenient, respectively. We also reported that time conflict with vaccination service and inconvenient transport to POVs were associated with a significantly reduced odds of vaccinating children against influenza. Targeted interventions are necessary, including extending vaccination service hours and an increase in the number of POVs close to residential areas.

Caregivers educated to bachelor’s degree level or above were more likely to vaccinate their children against influenza than people with lower educational attainment. Previous studies have shown that higher education may be associated with both lower and higher levels of vaccine acceptance [49]. Meanwhile, studies conducted in China showed that caregivers with higher education had a significantly higher odds of being reluctant acceptors [50]. We also found that people with the highest household income levels were less likely to vaccinate their children against influenza. Public health information campaigns need to be appropriately targeted and tailored to wealthier and less educated caregivers with the aim to provide them with professional and accurate vaccination information.

This study has several limitations. First, since we only included caregivers in three provinces, findings cannot be generalized to all provinces or districts in China. Second, the questionnaires were self-administered, which may have led to a degree of recall bias. Third, because around half the participants were recruited via POVs, there may be some selection bias resulting from our sampling methodology. Caregivers who take children to POVs may have higher vaccine confidence than those who do not present at POVs. Finally, the cross-sectional study design limits causal inference on the various factors observed.

## 5. Conclusions

Our study presents a higher childhood influenza vaccination uptake rate than previous Chinese studies. Caregivers who had a high confidence in influenza vaccines saw other caregivers vaccinating their children and positively supported vaccination from HCWs, family members, or friends, and had an increased odds of vaccinating their own children. Poor access, including conflicts between caregivers’ schedules and vaccination service and inconvenient transportation to POV, hinders the vaccination uptake. In addition to public health information campaigns to promote childhood influenza vaccination and enhance caregivers’ confidence, interventions to optimize access to vaccination services are needed. Interventions are needed to support encourage primary care providers to play a greater role in promoting vaccination. The dissemination of related information and the public response online need to be monitored for the timely understanding of public perceptions.

## Figures and Tables

**Table 1:** Determinants of childhood influenza vaccination.

Variables	Influenza Vaccination in 2020–2021 Flu Season		Univariate Analyses	Multivariate Analyses
	Vaccinated (%)	Unvaccinated (%)	*p*	OR
Total	908 (43.63)	1173 (56.37)	—	—
Region			0.001	
Anhui province	398 (48.24)	427 (51.76)		Ref.
Shenzhen city	134 (37.22)	226 (62.78)		0.8 (0.51–1.26)
Shaanxi province	376 (41.96)	520 (58.04)		0.66 (0.48–0.92) *
Living area			0.019	
Urban	592 (45.61)	706 (54.39)		Ref.
Rural	316 (40.36)	467 (59.64)		0.75 (0.47–1.20)
Registered residence			0.236	
Local residents	750 (44.25)	945 (55.75)		Ref.
Internal migrants	158 (40.93)	228 (59.07)		0.87 (0.69–1.10)
Caregiver’s age group (years)			0.731	
<=30	247 (44.83)	304 (55.17)		Ref.
~35	388 (44.09)	492 (55.91)		0.95 (0.68–1.33)
~40	164 (42.71)	220 (57.29)		0.94 (0.74–1.18)
>40	109 (40.98)	157 (59.02)		0.71 (0.36–1.39)
Gender (Caregiver)			0.106	
Female	715 (42.76)	957 (57.24)		Ref.
Male	193 (47.19)	216 (52.81)		1.22 (0.90–1.65)
Caregiver relationship with children			0.19	
Mother	691 (42.63)	930 (57.37)		Ref.
Father	153 (46.36)	177 (53.64)		0.89 (0.72–1.09)
Grandparents and others	64 (49.23)	66 (50.77)		1.79 (0.70–4.59)
Education			0.003	
Middle school or below	189 (37.72)	312 (62.28)		Ref.
High school	185 (41.39)	262 (58.61)		1.09 (0.89–1.33)
Junior college	250 (46.55)	287 (53.45)		1.32 (0.95–1.84)
Bachelor degree or above	284 (47.65)	312 (52.35)		1.41 (1.13–1.76) **
Annual household income (1000 Renminbi)			0.304	
<20	172 (40.38)	254 (59.62)		Ref.
20–50	172 (43.11)	227 (56.89)		0.99 (0.74–1.34)
50–100	253 (46.85)	287 (53.15)		0.97 (0.86–1.08)
100–200	196 (44.65)	243 (55.35)		0.81 (0.68–0.95) *
>200	115 (41.52)	162 (58.48)		0.73 (0.65–0.82) **
Gender (Child)			0.202	
Female	449 (45.08)	547 (54.92)		Ref.
Male	459 (42.3)	626 (57.7)		0.91 (0.79–1.04)
Child’s age group (months)			<0.001	
<24	222 (52.11)	204 (47.89)		Ref.
>=24	686 (41.45)	969 (58.55)		0.78 (0.54–1.11)
Susceptibility of flu			<0.001	
Low	146 (33.72)	287 (66.28)		Ref.
High	762 (46.24)	886 (53.76)		1.1 (0.70–1.73)
Susceptibility of flu during COVID-19			0.004	
Decreased	296 (39.47)	454 (60.53)		Ref.
Increased	612 (45.98)	719 (54.02)		0.87 (0.75–1.02)
Severity of flu			<0.001	
Low	261 (36.81)	448 (63.19)		Ref.
High	647 (47.16)	725 (52.84)		1.16 (0.92–1.47)
Confidence in flu vaccine effectiveness			<0.001	
Disagree	133 (25.58)	387 (74.42)		Ref.
Agree	775 (49.65)	786 (50.35)		1.54 (1.23–1.93) **
Confidence in flu vaccine importance			<0.001	
Disagree	48 (15.14)	269 (84.86)		Ref.
Agree	860 (48.75)	904 (51.25)		2.50 (1.77–3.54) **
Confidence in flu vaccine safety			<0.001	
Disagree	64 (20.13)	254 (79.87)		Ref.
Agree	844 (47.87)	919 (52.13)		1.60 (1.29–1.99) **
Time conflict with vaccination service			0.007	
Disagree	828 (44.66)	1026 (55.34)		Ref.
Agree	80 (35.24)	147 (64.76)		0.61 (0.40–0.92) *
Influenza vaccine is out of stock			0.428	
Disagree	714 (43.19)	939 (56.81)		Ref.
Agree	194 (45.33)	234 (54.67)		0.92 (0.73–1.17)
Influenza vaccine is expensive			0.091	
Disagree	754 (44.51)	940 (55.49)		Ref.
Agree	154 (39.79)	233 (60.21)		1.02 (0.66–1.56)
Unsatisfied with past vaccination service			0.475	
Disagree	820 (43.39)	1070 (56.61)		Ref.
Agree	88 (46.07)	103 (53.93)		1.4 (0.82–2.39)
Transport to POVs is inconvenient			0.047	
Disagree	836 (44.33)	1050 (55.67)		Ref.
Agree	72 (36.92)	123 (63.08)		0.66 (0.51–0.85) **
Most caregivers vaccinate their children			<0.001	
Disagree	371 (33.13)	749 (66.88)		Ref.
Agree	537 (55.88)	424 (44.12)		2.16 (1.94–2.40) **
Child’s self-rated health is poor			0.757	
Disagree	718 (43.81)	921 (56.19)		Ref.
Agree	190 (42.99)	252 (57.01)		0.79 (0.55–1.12)
Influence from HCWs			<0.001	
Negative	512 (38.47)	819 (61.53)		Ref.
Positive	396 (52.8)	354 (47.2)		1.33 (1.00–1.77) *
Influence from family members or friends			<0.001	
Negative	556 (38.8)	877 (61.2)		Ref.
Positive	352 (54.32)	296 (45.68)		1.30 (1.14–1.49) **
Influence from government departments			0.002	
Negative	562 (41.23)	801 (58.77)		Ref.
Positive	346 (48.19)	372 (51.81)		0.9 (0.58–1.38)
Influence from internet			<0.001	
Negative	732 (41.73)	1022 (58.27)		Ref.
Positive	176 (53.82)	151 (46.18)		1.04 (0.64–1.67)
Anxiety associated with flu vaccination			<0.001	
Yes	680 (41.36)	964 (58.64)		Ref.
No	228 (52.17)	209 (47.83)		1.74 (0.95–3.20)
Anxiety associated with not getting flu vaccination			0.145	
Yes	735 (44.44)	919 (55.56)		Ref.
No	173 (40.52)	254 (59.48)		0.64 (0.40–1.04)

Notes: Odds ratio and 95% confidence intervals were presented. Significance level: ** *p* < 0.01, * *p* <0.05.

## Data Availability

The corresponding author had full access to all the data in the study and had final responsibility for the decision to submit for publication.

## Appendix A

**Table A1:** Construction and corresponding questions used to measure the perception of influenza vaccine and disease and cue to action of caregivers.

Constructs	Question	Scale
Perceived susceptibility	How likely is your child to get the flu this fall or winter?	Completely possible, possible, neutral/not sure, impossible, completely impossible.
Perceived severity	If your child has the flu, what do you think the chances are of serious consequences (severe complications such as pneumonia)?	
Impact of COVID-19 on perceived susceptibility	Is there any change in children’s risk of influenza during COVID-19 compared to previous autumn and winter?	The risk becomes very high, risk increased, neutral/Not sure, risk reduced, the risk becomes very low.
Perceived benefit	Do you think vaccinating children against influenza is effective in preventing infection?	Definitely yes, possibly yes, not sure, possibly no, definitely no.
Perceived barrier (influenza vaccine importance)	Influenza vaccines are important for children to have.	Strongly disagree, disagree, neither agree nor disagree, agree, strongly agree.
Perceived barrier (influenza vaccine safety)	Overall, I think influenza vaccines are safe.	
Perceived barrier (accessibility)	Time for vaccination service conflicts with working time.	
Perceived barrier (availability)	There is a lack of availability of the influenza vaccine in my local hospital.	
Perceived barrier (acceptability)	Not satisfied with past vaccination service/healthcare service.	
Perceived barrier (affordability)	Influenza vaccine costs too much.	
Perceived barrier (accommodation)	Transportation to the POV is not very convenient.	
Cue to action	Most of the parents you know take their children for flu shots.	
	My child catching colds frequently.	

## Appendix B

**Table A2:** Respondent Characteristics, N (%).

Characteristics	Total Sample, N (%)
Region	
Anhui province	825 (39.64%)
Shenzhen city	360 (17.29%)
Shaanxi province	896 (43.06%)
Living area	
Urban	1298 (62.37%)
Rural	783 (37.63%)
Registered residence	
Local residents	1695 (81.45%)
Internal migrants	386 (18.55%)
Caregiver’s age group (years)	
<=30	551 (26.48%)
~35	880 (42.29%)
~40	384 (18.45%)
>40	266 (12.78%)
Gender (Caregiver)	
Female	1672 (80.35%)
Male	409 (19.65%)
Caregiver relationship with children	
Mother	1621 (77.9%)
Father	330 (15.86%)
Grandparents and others	130 (6.25%)
Education	
Middle school or below	501 (24.07%)
High school	447 (21.48%)
Junior college	537 (25.8%)
Bachelor degree or above	596 (28.64%)
Annual household income (1000 Renminbi)	
<20	426 (20.47%)
20–50	399 (19.17%)
50–100	540 (25.95%)
100–200	439 (21.1%)
>200	277 (13.31%)
Gender (Child)	
Female	996 (47.86%)
Male	1085 (52.14%)
Child’s age group (months)	
<24	426 (20.47%)
>=24	1655 (79.53%)
Susceptibility of flu	
Low	433 (20.81%)
High	1648 (79.19%)
Susceptibility of flu during COVID-19	
Decreased	750 (36.04%)
Increased	1331 (63.96%)
Severity of flu	
Low	709 (34.07%)
High	1372 (65.93%)
Confidence in flu vaccine effectiveness	
Disagree	520 (24.99%)
Agree	1561 (75.01%)
Confidence in flu vaccine importance	
Disagree	317 (15.23%)
Agree	1764 (84.77%)
Confidence in flu vaccine safety	
Disagree	318 (15.28%)
Agree	1763 (84.72%)
Time conflict with vaccination service	
Disagree	1854 (89.09%)
Agree	227 (10.91%)
Influenza vaccine is out of stock	
Disagree	1653 (79.43%)
Agree	428 (20.57%)
Influenza vaccine is expensive	
Disagree	1694 (81.4%)
Agree	387 (18.6%)
Unsatisfied with past vaccination service	
Disagree	1890 (90.82%)
Agree	191 (9.18%)
Transport to POVs is inconvenient	
Disagree	1886 (90.63%)
Agree	195 (9.37%)
Most caregivers vaccinate their children	
Disagree	1120 (53.82%)
Agree	961 (46.18%)
Child’s self-rated health is poor	
Disagree	1639 (78.76%)
Agree	442 (21.24%)
Influence from HCWs	
Negative	1331 (63.96%)
Positive	750 (36.04%)
Influence from family members or friends	
Negative	1433 (68.86%)
Positive	648 (31.14%)
Influence from government departments	
Negative	1363 (65.5%)
Positive	718 (34.5%)
Influence from internet	
Negative	1754 (84.29%)
Positive	327 (15.71%)
Anxiety associated with flu vaccination	
Yes	1644 (79%)
No	437 (21%)
Anxiety associated with not getting flu vaccination	
Yes	1654 (79.48%)
No	427 (20.52%)
